# Road Marking Distress Detection and Assessment Based on UAV Imagery

**DOI:** 10.3390/ma19050992

**Published:** 2026-03-04

**Authors:** Yunfan Nie, Wangjie Wu, Jinhuan Shan, Hongxin Peng, Feiyang Guo, Yaohan Liu, Jingjing Xiao

**Affiliations:** 1 School of Highway, Chang’an University, South 2nd Ring Road Middle Section, Xi’an 710064, China; 2024137006@chd.edu.cn (Y.N.); hongxin_peng@chd.edu.cn (H.P.); 2024021063@chd.edu.cn (F.G.); yaohanliu@chd.edu.cn (Y.L.); 2 School of Materials Science and Engineering, Chang’an University, South 2nd Ring Road Middle Section, Xi’an 710064, China; wwj@chd.edu.cn; 3 School of Civil Engineering, Chang’an University, South 2nd Ring Road Middle Section, Xi’an 710064, China

**Keywords:** UAV, road markings, distress detection, distress ratio calculation, image matching

## Abstract

With the continuous advancement of autonomous driving technology, lane marking-based environment perception has become a critical component of autonomous vehicle systems. However, long-term vehicle loads cause road markings to deteriorate and fade, significantly compromising driving safety. Traditional road marking quality inspection methods are inefficient and struggle to achieve high-performance, convenient detection. To address these challenges, this paper proposes an integrated framework for road marking detection and evaluation using Unmanned Aerial Vehicle (UAV) imagery. The framework comprises three core modules: lightweight data acquisition, efficient marking extraction, and accurate distress assessment. First, optimized UAV flight parameters enable low-cost, highly flexible, and safe data collection. Second, the YOLOv8-MEB model, combined with instance segmentation screening and local image optimization, achieves lane segmentation precision and recall above 90% with FPS exceeding 60. Furthermore, a standard marking template library is constructed, and a RANSAC-based template matching method with affine transformation is employed to restore intact marking shapes. A contour correction strategy is introduced to mitigate errors induced by construction inaccuracies. The proposed framework supports nine common types of road markings and yields approximately 10% error in distress ratio calculation under non-severe damage conditions, providing a practical technical reference for intelligent road maintenance.

## 1. Introduction

As a critical component of traffic safety facilities, road markings play a vital role in guiding drivers and maintaining traffic order [1,2]. Studies have shown that clear and intact road markings can significantly reduce traffic accident rates and strongly support traffic safety [3]. With continuous technological advancements, autonomous driving has become a major development direction in modern transportation. Among its key technologies, the automatic recognition of road markings relies heavily on their completeness and accuracy, which directly affects the reliability of autonomous driving systems [4,5]. However, asphalt pavement is prone to fatigue damage due to long-term exposure to natural environments and frequent vehicle loads, leading to issues such as damage, breakage, blurred edges, and fading of road markings. This not only impairs drivers’ recognition and judgment but also significantly increases the risk of traffic accidents [6,7]. According to the US public datasets (CISS and NASS-CDS), traffic accidents caused by the combined effects of road infrastructure and environment account for 45% of the total number of traffic accidents [8]. In particular, the service life of commonly used thermoplastic marking materials generally does not exceed three years under normal conditions, averaging only one to two years, implying that markings often require multiple rounds of maintenance and renewal throughout a road’s lifecycle [9]. Therefore, regular inspection and maintenance of road markings are particularly important.

Traditional road marking inspection primarily relies on manual methods, where professional inspectors conduct visual examinations along target road sections, often supplemented by specialized equipment like retroreflectometers to measure optical performance indicators such as retroreflectivity [10]. Such equipment is expensive and requires professional operation, while manual approaches face limitations in efficiency, accuracy, and safety, especially when measuring irregular damaged areas with rulers, leading to significant human errors and inconsistencies, with permissible error rates for damaged areas as high as 25% [11]. In recent years, dedicated road inspection vehicles have improved efficiency and safety; as early as the 1980s, researchers mounted cameras on vehicles to develop automated crack monitoring systems using videotapes [12]. To address limitations of 2D images susceptible to environmental variations, 3D sensors like lasers, LiDAR, and stereo cameras have been integrated [13,14,15,16]. Despite these advances, such vehicles remain costly and complex, limiting their deployment to critical road sections or high-grade highways.

With the rapid development of unmanned aerial vehicle (UAV) technology, its potential has been demonstrated in road infrastructure monitoring, offering operational flexibility, low cost, and the ability to overcome terrain restrictions [17,18,19]. UAVs enable multi-angle, close-range image acquisition and automated tasks, effectively surpassing conventional methods [20,21]. Current research has focused on road marking or lane line detection [22,23], with lane detection aiding vehicle positioning in autonomous driving [24]. In inspections, studies identify marking integrity to locate defects but often lack in-depth condition evaluation [25,26]. Deep learning has enabled high-precision detection [27], yet model performance depends on high-quality data, posing ongoing challenges.

Recognition and classification of road markings traditionally rely on morphological and optical characteristics [28]. Early methods used features like MSER and template matching for detection from in-vehicle videos [29], classifying contours based on shapes for specific types [30,31]. Color information has been combined with shape priors to handle poor lighting and damaged markings [32], while metrics like correctness, shape, and visibility have been proposed for lane quality evaluation using dual-modal algorithms [33]. Deep learning advancements, particularly the YOLO series, have enhanced efficiency through attention mechanisms, optimized architectures, and lightweight models [34,35,36,37]. Object detection and semantic segmentation are commonly employed [38,39], with hybrid approaches addressing contextual loss but at the cost of efficiency [40]. Convolutional neural networks have recognized damaged arrow markings, though without effective damage degree assessment [41].

For damage assessment, average width after threshold segmentation serves as an indicator [42,43], and YOLOv4 combined with U-Net has used grayscale values for preliminary evaluation [44]. However, grayscale variations under different lighting limit applicability. Overall, research emphasizes algorithm optimization or specific damage recognition, lacking a comprehensive system for data acquisition, extraction, restoration, and quantification.

**Figure 1:**
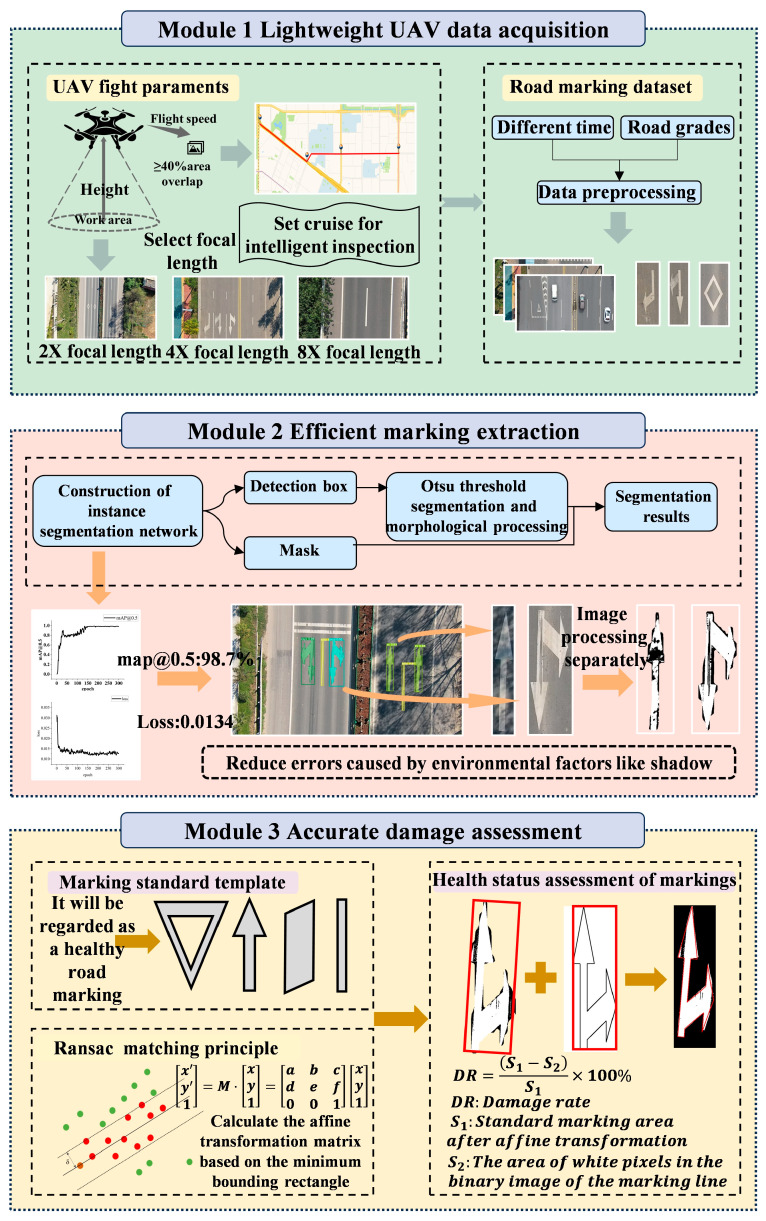
Framework flow chart.

Compared to traditional manual and vehicle-based methods, UAV-based inspection offers efficiency and cost advantages for marking distress assessment, providing wide coverage and suitability for large-scale networks. This paper proposes a comprehensive UAV-based evaluation framework for road markings, achieving a closed-loop pipeline from lightweight data acquisition and efficient marking extraction to precise distress assessment, as illustrated in Figure 1. The framework establishes balanced flight parameters to overcome cost and geographical constraints, designs a two-stage workflow for robustness against illumination variations, introduces dynamic template matching and contour correction for accurate quantitative assessment across multiple marking types, and validates its effectiveness through experimental analyses.

## 2. Methodology

### 2.1. Lightweight UAV Data Acquisition

#### 2.1.1. Determination of UAV Flight Parameters

UAVs provide low cost, operational convenience, and intelligent capabilities, offering clear advantages over traditional vehicle-mounted systems and serving as a vital tool for lightweight data collection. In this study, a DJI Matrice 300 RTK quadcopter drone equipped with a Zenmuse H20N gimbal camera was used for road image data collection. The specific parameters of the equipment are shown in Table 1. In the field of road disease detection, compared to conventional drone models (such as the DJI Mavic 3), this equipment has excellent high-altitude hovering, cruising, and urban airflow resistance capabilities. Coupled with a 20× optical zoom lens, it can accurately capture road surface details at high altitudes. However, due to constraints on flight height and sensor size, it has a relatively large Ground Sampling Distance (GSD) and weaker detection performance for subtle diseases (such as cracks with a width of 1 mm). For image data collection, the flight altitude of the UAV and the selection of the camera lens focal length are crucial factors significantly influencing data quality. Determining appropriate flight height and lens parameters is therefore essential, as illustrated in Figure 1.

**Figure 2:**
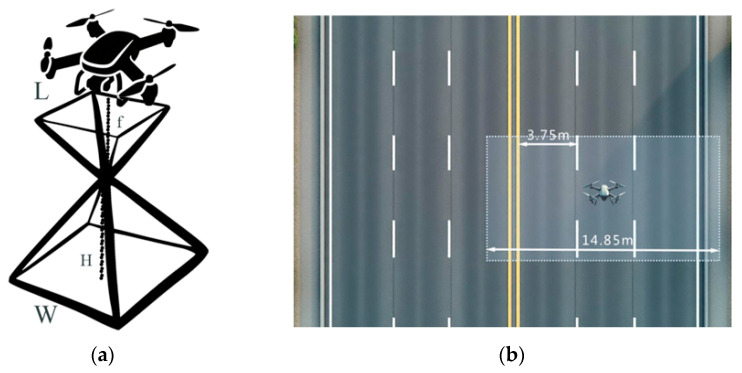
Schematic diagram of drone flight. (**a**) Schematic diagram of drone’s field of view; (**b**) Schematic diagram of drone visual coverage area.

In this study, an orthographic projection method was employed for road data acquisition using a UAV. The UAV was flown along the central axis on one side of the road in a single direction, capable of simultaneously covering up to three lanes in one pass. The camera field of view (FOV) in this study refers to the area captured by the UAV-mounted camera. When determining flight parameters, it is essential to ensure that the camera FOV fully covers the target road surface. Wide-angle lenses exhibit certain geometric distortions, particularly in the outer 10–15% of the image area, where reduced image quality is often observed. Therefore, when using a wide-angle lens for image capture, it is advisable to utilize the central region of the FOV as much as possible. The camera FOV follows the pinhole imaging model, as shown in Figure 2 and Equation (1), where *W* represents the horizontal and vertical dimensions of the camera FOV, *H* denotes the operating height of the camera, *L* corresponds to the size of the camera sensor, and *f* is the focal length of the camera lens.

(1) $$W=\frac{H\times L}{f}$$

Urban environments contain numerous transportation infrastructure elements and low-rise buildings. To ensure both traffic safety and UAV operational safety, the minimum flight altitude during data acquisition was set no lower than 30 m. Additionally, to prevent the truncation of continuous road markings during data collection, the longitudinal length of the camera field of view should be no less than 10 m. Table 2 presents the minimum flight altitudes required for different numbers of lanes and various lens focal lengths. To guarantee image quality, the outermost 10% of the image area is discarded.

To ensure the integrity of road data, the overlap rate between adjacent images during UAV data acquisition should be no less than 40%. Moreover, unmanned aerial vehicles are prone to motion blur in images during flight due to their fast flight speed and high wind speed, manifested as pixel displacement in the direction of motion. This will seriously affect the quality of the image and ultimately lead to a decline in model performance. In order to prevent poor image quality from affecting subsequent experiments, it is necessary to control the exposure parameters and flight speed of the camera in conjunction with the daily environment during the data acquisition stage to reduce the impact of motion blur. Considering the urban operational context, and after comprehensive evaluation of factors such as camera focal length, the minimum safe flight altitude in urban traffic environments, and the minimum required longitudinal field of view length, this study ultimately selected the following parameters for data acquisition: 4× optical zoom (24.25 mm), a flight altitude of 50 m, and a flight speed of 5 m/s, as illustrated in Figure 2.

#### 2.1.2. Data Preprocessing

**Figure 3:**
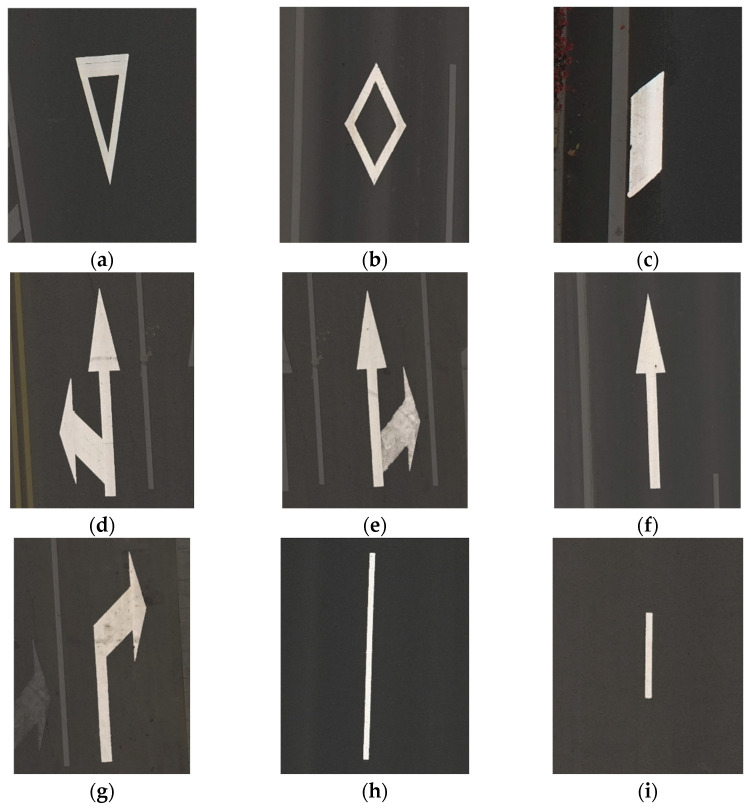
Road marking definition diagram (Highlight the corresponding markings). (**a**) Deceleration yield; (**b**) Zebra crossing warning; (**c**) Straight deceleration; (**d**) Straight left turn; (**e**) Straight right turn; (**f**) Straight line; (**g**) Right turn; (**h**) Long lane boundary; (**i**) Short lane boundary.

In this study, road marking videos were captured using a UAV under varying lighting conditions on roads of different classes in Xi’an, China, at a resolution of 2688 × 1512. After screening over 20,000 images, a dataset containing 2648 images was ultimately constructed. Due to the widespread distortion of camera lenses, images can be corrected by using calibrated camera parameters. Directly using uncorrected images will not only affect the generalization of the model (especially wide-angle lenses), but also affect the subsequent area calculation work. The dataset defines a total of nine distinct types of road markings, as shown in Figure 3.

In order to improve the robustness of the dataset under complex real-world conditions and the adaptability of the model, we applied data augmentation techniques, including scaling, rotation, and adding noise. And some images with shadows are retained in the dataset to simulate the possible situations that may occur during the conventional acquisition process. Among them, motion blur will reduce edge clarity, thereby lowering the segmentation accuracy and recall rate of detail labeling. Shadows can cause inconsistent brightness, making it easy to identify shaded areas as road surfaces, which can increase false positives and misclassification in instance segmentation. The dataset was randomly divided into training (70%), validation (20%), and testing (10%) subsets.

#### 2.1.3. Data Annotation

YOLO is a highly optimized CNN architecture that employs a supervised learning approach, requiring manual annotation of the dataset prior to training to guide the learning process. In this study, Labelme was used to annotate the dataset. The annotation files were stored in JSON format, containing information such as marking categories and coordinates. After completing the annotation process, all JSON files were converted to TXT format for easier processing. To ensure the quality of the annotation files, the following requirements were strictly followed during the Labelme annotation process:

- Due to lens distortion, road markings located at the edges of the images were not annotated.
- Markings that were incomplete due to occlusion, significant shadows, or severe damage were not annotated.

### 2.2. Efficient Road Marking Extraction

#### 2.2.1. Instance Segmentation Model

**Figure 4:**
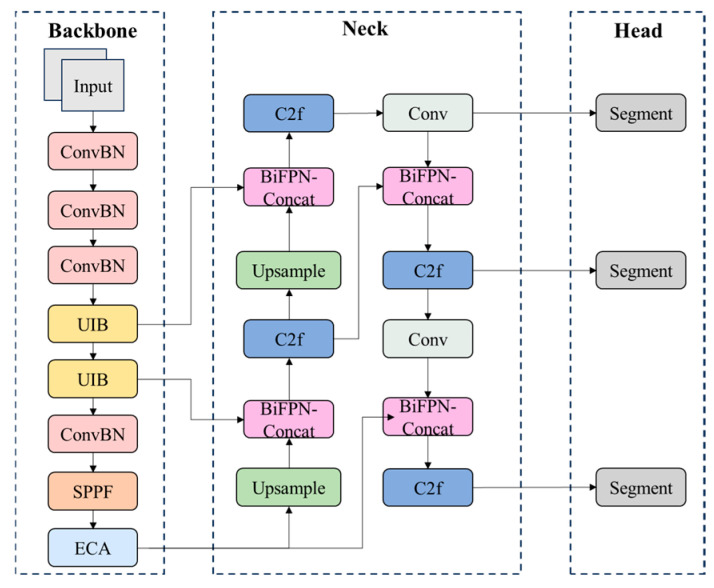
YOLOv8-MEB network architecture.

Considering the characteristics of road marking targets, such as significant size variations, diverse shapes, and complex background interference, this paper constructs the YOLOv8-MEB model based on YOLOv8, as illustrated in Figure 4. This model retains the original end-to-end inference advantage while designing the backbone feature extraction network and feature fusion structure. It enhances the model’s lightweight degree and feature expression efficiency while ensuring model accuracy.

**Figure 5:**
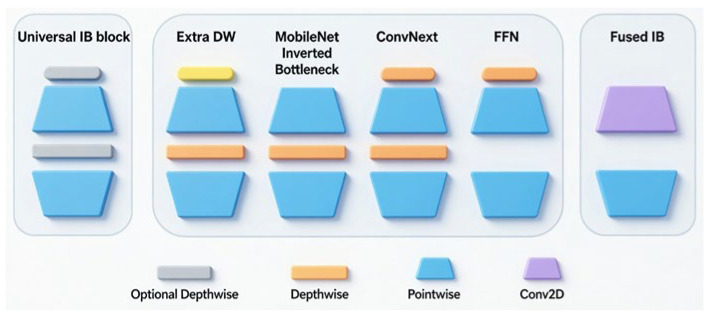
Universal Inverted Bottleneck (UIB) module in MobileNetV4 [45].

YOLOv8’s CSPDarkNet backbone offers strong feature extraction but high computational cost. We replaced it with MobileNetV4 [45], which uses the Universal Inverted Bottleneck (UIB) for better efficiency while maintaining feature quality Figure 5. A Spatial Pyramid Pooling Fast Module (SPPF) is introduced at the end of the backbone network to aggregate multi-scale contextual information and enhance the model’s receptive field coverage ability for different scale road markings. At the same time, an Efficient Channel Attention (ECA) mechanism is introduced after the SPPF output. By modeling channel importance without significantly increasing model complexity, it strengthens discriminative feature responses related to road markings and suppresses interference from complex road backgrounds and redundant features on high-level semantic representations.

**Figure 6:**
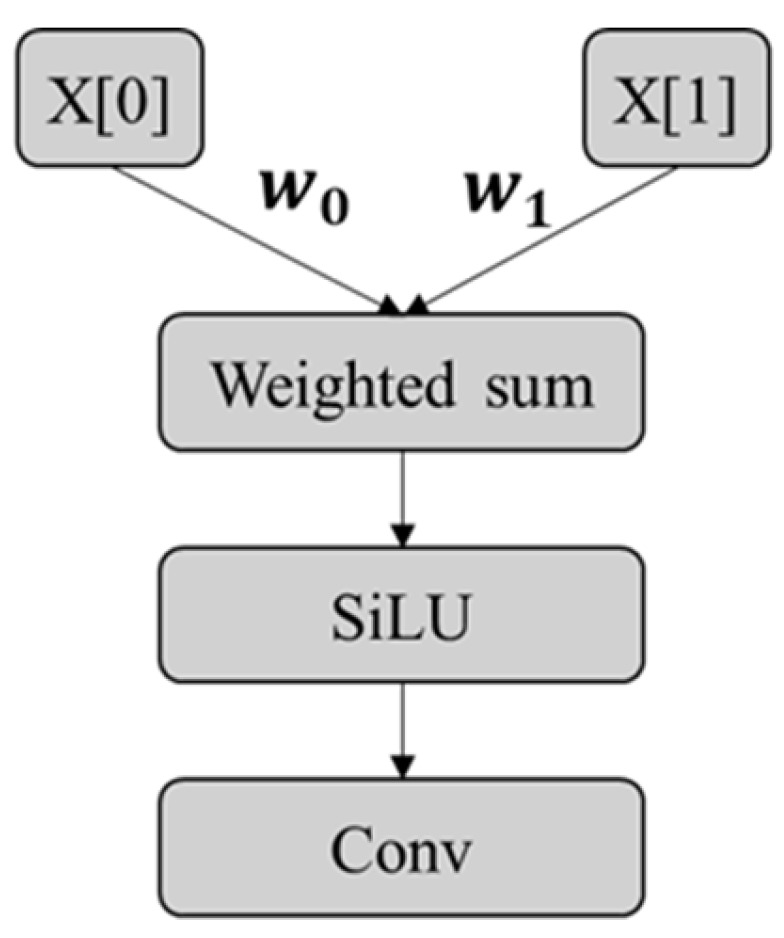
BiFPN-Concat structure.

In the feature fusion stage, this paper improves the original neck structure of YOLOv8 by introducing the fusion idea of the weighted Bi-directional Feature Pyramid Network (BiFPN), as shown in Figure 6. By constructing a feature fusion module based on BiFPN-Concat, it adaptively weights and concatenates features from different paths while maintaining consistent feature spatial scales, achieving a more balanced fusion expression of shallow-level detail features and deep-level semantic features at the same scale. This fusion method can effectively alleviate the problem of uneven contribution of features at different scales in traditional feature concatenation, which is beneficial for enhancing the instance segmentation model’s ability to depict road marking continuity, integrity, and boundary information.

The primary function of the loss function is to measure the discrepancy between the model’s predicted results and the actual labels. It typically consists of multiple components, including bounding box regression loss, confidence loss, and classification loss. In this study, the original bounding box regression loss function is replaced with Wise-IoU. Wise-IoU introduces an adaptive weight factor related to prediction quality. Its core idea is to dynamically adjust the contribution weight of regression loss based on the overlap between the predicted box and the ground truth box, allowing high-quality predicted samples to occupy a higher weight in the training process, while appropriately suppressing the impact of low-quality predicted samples on gradient updates.

In this study, experiments were conducted using the PyTorch (2.1.2) framework under the Windows operating system. The relevant parameters of the experimental environment are summarized in Table 3.

After model training is completed, precision, mAP (mean Average Precision), and recall are used to evaluate the performance of the trained model. Depending on the model type, prediction results can be categorized into four cases:

- True Positive (TP): The actual sample is true and the model prediction is correct.
- True Negative (TN): The sample is actually false, and the model prediction is correct.
- False Positive (FP): The sample is actually false, and the model prediction is wrong.
- False Positive (FN): The sample is actually true, and the model prediction is wrong.

#### 2.2.2. Image Tiling

Instance segmentation primarily aims to perform pixel-level segmentation of each target in an image, thereby obtaining precise locations and sizes of objects. Unlike traditional image threshold segmentation, it not only requires separating objects of different categories but also necessitates segmenting instances of the same category into distinct individual entities [46].

**Figure 7:**
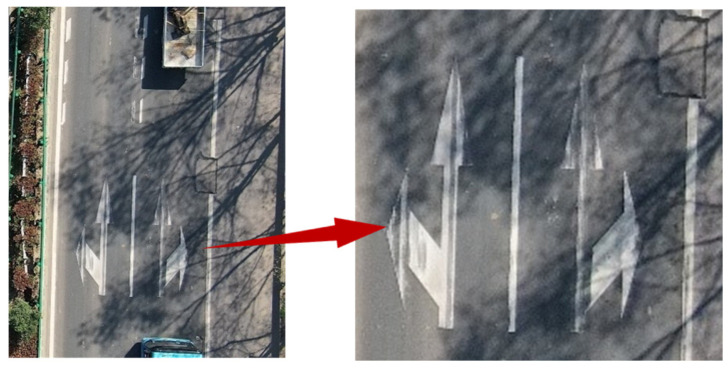
Pictures with shadows.

Threshold segmentation is a commonly used method for extracting road markings. However, due to potential local shadows, water accumulation, and occlusion on the road surface, as shown in Figure 7, the accuracy of threshold segmentation may be compromised by localized shadows or highlights. To mitigate this, the bounding box information of road markings obtained through instance segmentation is used to isolate the markings from the original image. The extracted markings are then stored and processed separately according to their categories. This approach effectively reduces the impact of abnormal conditions such as shadows and water accumulation on marking recognition, while allowing independent subsequent processing for each road marking.

#### 2.2.3. Road Marking Extraction

The fundamental principle of threshold segmentation lies in determining a threshold to divide an image into two parts: the target object and the background. This method is characterized by its simplicity and high operational efficiency. Due to the significant contrast between road markings and the pavement background, which allows for straightforward distinction, threshold segmentation is well-suited as an extraction technique. After evaluating various threshold segmentation methods, the Otsu threshold segmentation algorithm [47] was ultimately employed.

Assume an image has dimensions *M* × *N*, and a binarization threshold *k* divides the image into background and foreground regions. Let the number of pixels in the background be $N_{0}$, and in the foreground be $N_{1}$. The proportion of background pixels is denoted as $w_{0}$, with a mean grayscale value of $u_{0}$; the proportion of foreground pixels is $w_{1}$, with a mean grayscale value of $u_{1}$; and the global mean grayscale value is *u*. The inter-class variance is calculated as shown in Equation (2). By iterating through all possible thresholds *k*, the corresponding inter-class variance *g* is computed. The optimal threshold *k* is determined when *g* is maximized. Here, $X_{1}$ represents the grayscale values of the background, and $X_{2}$ represents the grayscale values of the foreground.

(2) $$g=w_{0}\times {\left(u_{0}-u\right)}^{2}+w_{1}\times {\left(u_{1}-u\right)}^{2}$$

(3) $$gw_{0}=\frac{N_{0}}{\left(N_{0}+N_{1}\right)}$$

(4) $$w_{1}=\frac{N_{1}}{\left(N_{0}+N_{1}\right)}$$

(5) $$gu_{0}=\frac{\sum X_{1}}{N_{0}}$$

(6) $$gu_{1}=\frac{\sum X_{2}}{N_{1}}$$

(7) $$u=\frac{(\sum X_{1}+\sum X_{2})}{\left(N_{0}+N_{1}\right)}$$

After simplification and substitution, another form of *g* is obtained:

(8) $$g=w_{0}\times w_{1}\times {\left(u_{0}-u_{1}\right)}^{2}$$

Since the obtained binarized road marking images contain noise that may affect subsequent processing, the opening operation in morphological processing methods can effectively address this issue. The opening operation refers to a process where the image first undergoes erosion followed by dilation, which helps remove noise and smooth the contours.

### 2.3. Calculation of Road Marking Damage

#### 2.3.1. Establishment of a Standard Road Marking Library

Road marking damage typically progresses from the exterior inward, eventually leading to contour loss and fragmentation. Even if the current condition and area of the markings are obtained through extraction methods, the lack of intact contours makes it difficult to determine their original area and positional information, thereby hindering quantitative assessment of their health status.

**Figure 8:**
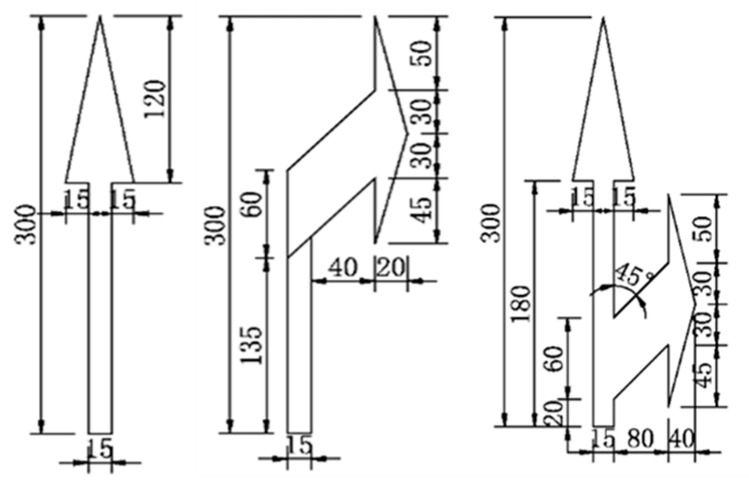
Marking standard template [48].

By referring to relevant road marking design standards, standard drawings of road markings were created to serve as references for their intact conditions. While design standards vary for different design speeds (typically categorized as below 40 km/h and above 40 km/h), the dimensions of the same type of marking at low and high design speeds are proportionally scaled by a factor of two. Therefore, the same standard drawing can be used as a reference regardless of design speed differences. However, variations in road classifications may still lead to differences in local marking dimensions. During detection, it is necessary to use corresponding standard marking templates based on specific road classifications. For example, Figure 8 shows some marking templates designed for a speed of 40 km/h.

#### 2.3.2. Image Matching

The RANSAC algorithm [49] is a robust method for model parameter estimation. Its underlying principle assumes that the sample data contains both inliers (correct data) and outliers (abnormal data), which may arise from erroneous measurements, invalid assumptions, or parameter calculation errors. In this context, outliers correspond to road markings with damaged contours and errors introduced during processing, while the standard marking template represents the ideal data.

The core idea of RANSAC is to estimate model parameters and evaluate their consistency by iteratively randomly sampling minimal subsets. Specifically, in each iteration, the algorithm randomly selects the minimal required data points to compute candidate model parameters. The remaining data points are then classified as inliers or outliers based on a predefined distance threshold. The number of inliers supporting the candidate model is recorded. After a predetermined number of iterations or upon meeting a stopping criterion, the model with the highest number of inliers is selected as the optimal estimate. The final model is typically refined using all inliers.

The minimum bounding rectangle of a shape effectively captures its position and size information. The fundamental attributes of a shape can be determined using only the center point and the two endpoints of its minimum bounding rectangle.

Affine transformation is a geometric transformation in two-dimensional or three-dimensional space that preserves the collinearity of points and parallelism of lines. Mathematically, it combines linear transformations (such as rotation, scaling, and shearing) with translation. In two-dimensional space, it can be expressed by Equation (9), where *M* represents the affine transformation matrix. Parameters *a*, *b*, *d*, and *e* control the linear transformation (e.g., rotation, scaling, shearing), while *c* and *f* control the translation components.

(9) $$\left[\begin{array}{c} x\prime \\ y\prime \\ 1 \end{array}\right]=M\cdot \left[\begin{array}{c} x\\ y\\ 1 \end{array}\right]=\left[\begin{array}{ccc} a & b & c\\ d & e & f\\ 0 & 0 & 1 \end{array}\right]\left[\begin{array}{c} x\\ y\\ 1 \end{array}\right]$$

**Figure 9:**
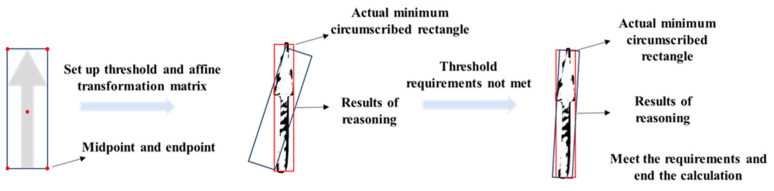
Calculation principle of RANSAC algorithm.

Traditional template matching methods require the presence of distinct similar features between two images. However, for damaged road markings, the lack of characteristic features makes it impossible to achieve accurate matching between standard marking templates and degraded markings using any conventional feature-based approach. For non-severely damaged markings, the minimum bounding rectangle serves as a significant image feature. The template matching method based on the RANSAC algorithm can treat discrete points along the damaged marking edges as outliers and effectively eliminate them, as illustrated in Figure 9. This process ultimately yields an appropriate affine transformation matrix to achieve accurate image alignment.

#### 2.3.3. Dynamic Contour Correction

The distress ratio is calculated as shown in Equation (10): *DR* = ($S_{1}$ − $S_{2}$)/$S_{1}$, where $S_{1}$ is the area of the transformed standard template (representing the intact marking), and $S_{2}$ is the area of white pixels in the extracted binarized image (representing the damaged marking).

(10) $$DR=\frac{\left(S_{1}-S_{2}\right)}{S_{1}}\times 100\%$$

**Figure 10:**
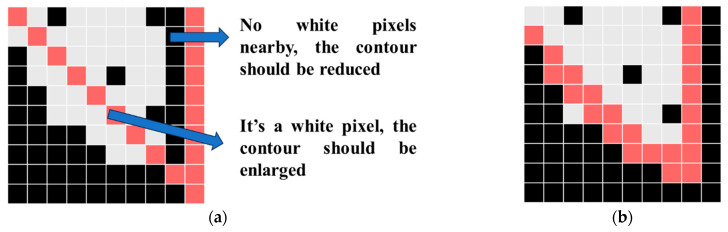
Standard marking contour correction. (**a**) Before correction; (**b**) After correction.

Since road markings may not be perfectly sprayed according to prescribed templates due to non-standardized operational practices during construction (e.g., over-spraying or under-spraying), a distress ratio correction method is proposed to more accurately calculate the actual distress ratio of markings. The correction process follows these steps:

1. Traverse each contour point of the standard marking template after affine transformation and process differently based on whether the pixel at the contour position is black or white.
2. For contour points where the corresponding pixel is black, if there are no white pixels within a one-pixel range in the surrounding area, contract the contour inward by one pixel, as illustrated in Figure 10.
3. For contour points where the corresponding pixel is white, expand the contour outward by one pixel. Expansion only occurs at white pixel positions and does not affect black pixels, ensuring that the expanded contour does not exceed boundaries.
4. Automatically connect the processed contour points in the nearest manner to form a closed new contour. The maximum outer contour of this new shape is then used to calculate the corrected area of the standard marking.

## 3. Results and Discussion

### 3.1. Performance of the Instance Segmentation Model

**Figure 11:**
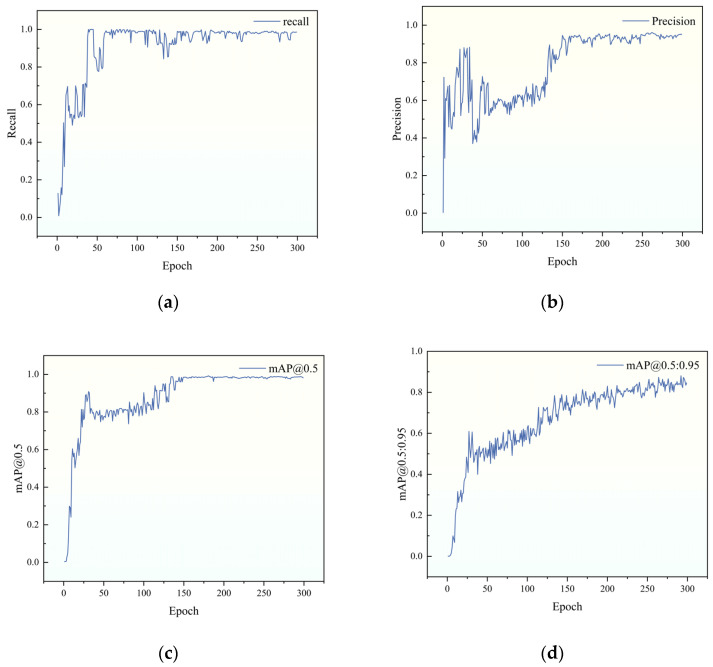
Model parameters. (**a**) Recall; (**b**) Precision; (**c**) mAP@0.5; (**d**) mAP@0.5:0.95.

During model training, accuracy is typically evaluated using metrics such as Recall, Precision, mAP@0.5, and mAP@0.5:0.95. As shown in Figure 11, if model accuracy is low, it can be improved by introducing higher-quality training data or increasing the number of training epochs. In this study, the model achieved optimal weights within the 50 to 150 epoch range, with a Recall of 0.983, Precision of 0.955, mAP@0.5 of 0.987, and mAP@0.5:0.95 of 0.873.

Figure 11 shows Precision and mAP@0.5 rising rapidly (0–50 epochs), briefly declining then stabilizing (50–150 epochs), with slight recovery in late training (150–250 epochs). This reflects early learning of salient features, mid-phase degradation from occlusion, lighting, and scene interference, and late-phase generalization.

However, mAP@0.5:0.95 did not follow the same trend but instead showed steady growth, indicating that the model prioritized localization refinement during the learning process rather than merely adjusting detection confidence.

To evaluate the effectiveness of the proposed network structure, we conducted ablation experiments to assess the impact of key components, and the results are shown in Table 4. The experimental results show that both ECA and BiFPN-Concat can improve the overall performance of the model on the basis of Mobilenetv4.

To ensure the fairness and rigor of the experiment, we tested our proposed model on the Ceymo dataset [50], which includes 11 types of road markings captured under challenging conditions (normal, crowded, glare, nighttime, rainfall, shadow), totaling 2887 images, totaling 2887 images.

**Figure 12:**
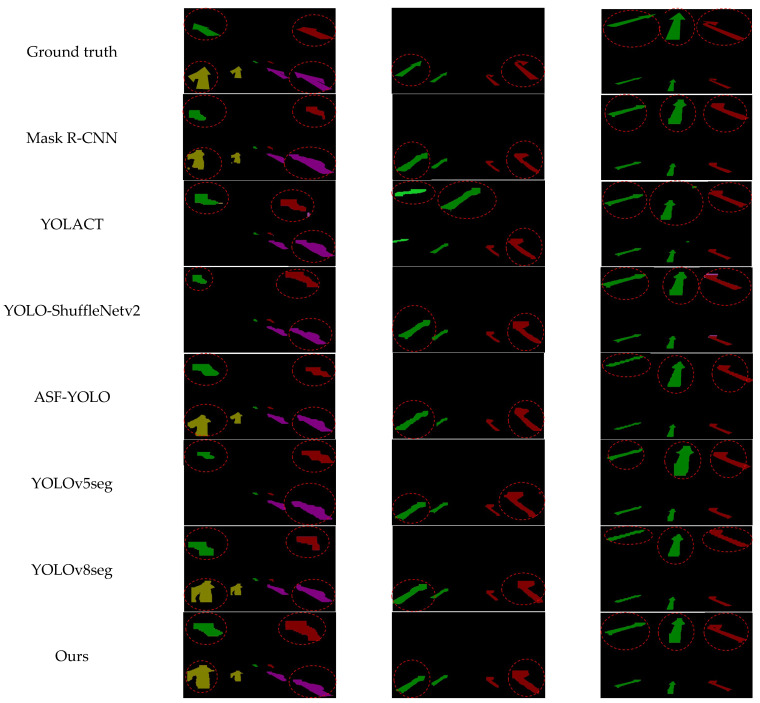
Inspection effect diagram.

The proposed YOLOv8-MEB model’s specific quantitative results with other models are shown in Table 5 and illustrated in Figure 12. This model achieves an actual inference speed of 72 FPS with 5 million parameters, while obtaining 86.1% box mAP and 85.2% mask mAP. This is because replacing the CSPDarkNet backbone with the MobileNetV4 architecture reduces computational cost, while introducing the ECA mechanism improves channel feature discrimination, and adopting the BiFPN weighted fusion strategy preserves the model’s detection accuracy as much as possible under the premise of reduced computational cost. These designs make it superior in accuracy to other lightweight models (e.g., YOLO-ShuffleNetv2 at 84.1%/83.8% and YOLOv5seg at 84.9%/84.1%), while maintaining real-time inference capability.

The core of the framework design is to enhance the computational speed for lightweight UAV deployment. In practical trade-offs, the model excels in runtime (72 FPS) and parameter efficiency (5 M), significantly surpassing more complex models such as Mask-R-CNN (8 FPS, 32 M parameters), ASF-YOLO (21 FPS, 46 M parameters), and YOLACT (15 FPS, 36 M parameters). Although there is a certain accuracy gap relative to the highest-accuracy models (e.g., ASF-YOLO at 89.5%/88.8% and YOLOv8seg at 86.9%/85.8%), the difference is small; considering its improvements in speed and deployability, this is an acceptable limitation. Overall, this model is suitable for lightweight devices with high requirements for lightweight and real-time performance, and has certain limitations for tasks with higher requirements for detection accuracy.

### 3.2. Accurate Extraction of Road Markings

**Figure 13:**
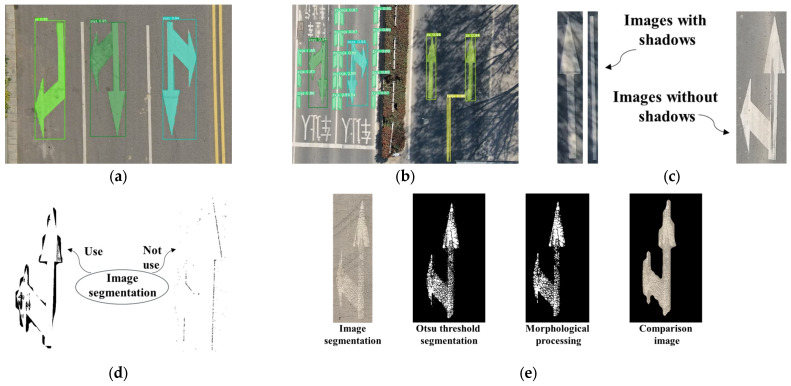
Road marking image extraction. (**a**) Instance segmentation results; (**b**) Detection result of existing shadow image; (**c**) Image segmentation example; (**d**) Whether to use the shadow image extraction results from image segmentation; (**e**) Line marking area extraction.

Instance segmentation processing of road images collected by UAVs yields result images as shown in Figure 13a, which include both detection bounding boxes and segmentation masks of the road markings. However, in cases where significant shadows are present, as illustrated in Figure 13b, global processing is susceptible to local interference, adversely affecting overall segmentation quality. To address this, this study adopts an image tiling strategy, dividing the image into multiple sub-regions to facilitate the classification and segmentation of different types of road markings. The results are demonstrated in Figure 13c. Taking the straight-and-left-turn combination marking in Figure 13b as an example, a comparison between the image tiling method and direct global threshold segmentation is shown in Figure 13d. It can be observed that although the target marking is not located in a shadowed area, traditional threshold segmentation incorrectly identifies parts of the shadow as background due to interference from shadows in the overall image, leading to segmentation deviations. In contrast, the image tiling method effectively suppresses such interference through localized processing, enhancing the independence of segmentation for each marking. After image tiling, the Otsu threshold segmentation method is applied to extract road markings within each sub-region. Taking the straight-and-left-turn marking in Figure 13a as an example, such markings are typically located on road sections with high traffic load and long service periods, often exhibiting degradation such as cracking, fragmentation, and fading of asphalt pavement. Although the Otsu algorithm demonstrates good adaptability in marking extraction, the results still contain certain noise and contour errors. To improve segmentation quality, this study further refines the Otsu segmentation results using the marking masks generated by instance segmentation, followed by morphological opening operations. This approach significantly eliminates noise interference and smoothens the marking contours, as shown in Figure 13e. Experimental results indicate that the proposed method maintains robust performance under complex road conditions such as shadows and water accumulation, effectively suppressing the impact of local image issues on overall processing quality.

**Figure 14:**
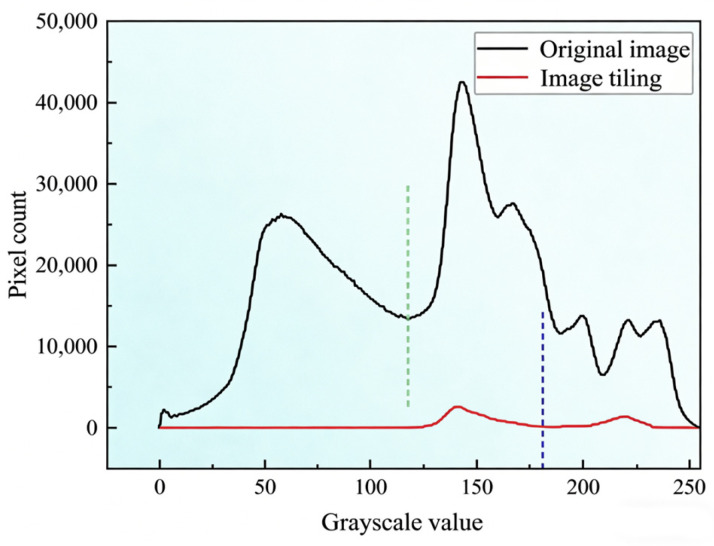
Analysis on Grayscale Values of Images Before and After Image Tiling Processing.

Pixel analysis was conducted on the road markings in Figure 13b, and threshold segmentation was performed separately on the image processed with image tiling and the original image. The relationship between pixel counts and grayscale values is presented in Figure 14, where the green dashed line (grayscale value = 121) indicates the segmentation threshold of the original image, and the blue dashed line (grayscale value = 181) represents that of the image after image tiling processing. This phenomenon arises because the shadows in the original image were misidentified as ground (the leftmost peak in the image), while the actual pavement was falsely recognized as road markings (the middle peak in the image). It can thus be concluded that image tiling exerts a positive effect on the processing of shadow-containing images.

### 3.3. Precise Assessment of Road Marking Damage

**Figure 15:**
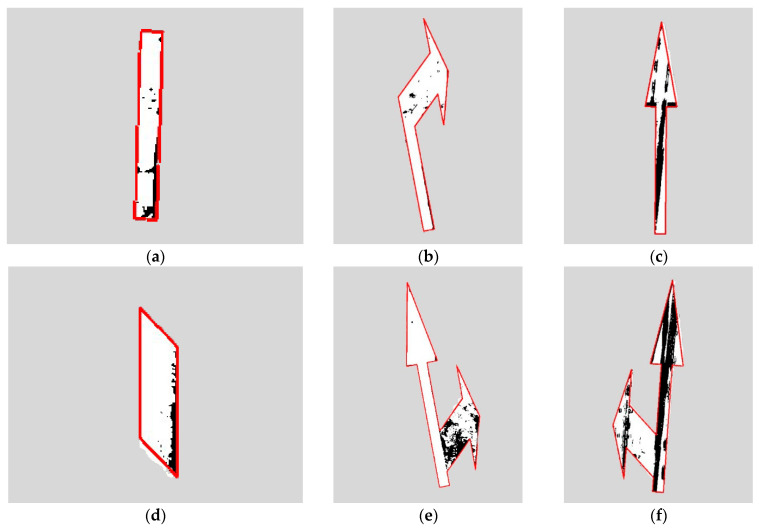
Road marking image matching. (**a**) Short lane boundary; (**b**) Right turn; (**c**) Straight line; (**d**) Straight deceleration; (**e**) Straight right turn; (**f**) Straight left turn.

The minimum bounding rectangle data of both the standard road marking template and the road markings extracted from real images are obtained. The RANSAC algorithm is then employed to compute the affine transformation matrix between these two minimum bounding rectangles. Subsequently, the standard marking template is transformed using this affine transformation matrix to achieve optimal alignment with the extracted road markings, representing the intact state of the road markings prior to damage. For visualization purposes, the contour of the transformed template is outlined in red, as shown in Figure 15.

Although the RANSAC-based template matching method achieves satisfactory alignment between the standard marking template and the actually extracted markings, and uses the matched template to represent the intact state of the marking, certain errors persist for some markings. As shown in Figure 15d, these errors typically arise due to construction inaccuracies during marking application, where the actual shape deviates from the standard template. Meanwhile, Figure 15f demonstrates errors caused by severe internal damage and significant loss of contour information, which adversely affect the matching process.

**Figure 16:**
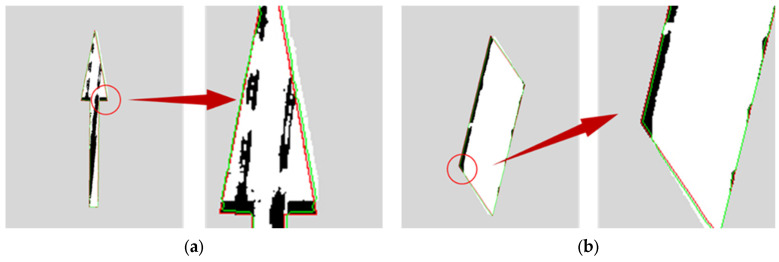
Contour correction result. (**a**) Description of what is contained in the first panel; (**b**) Contour correction of construction errors.

The distress ratio of the road markings shown in Figure 15 was calculated, and the differences before and after applying the corrected standard marking contour method were compared. The road marking contours before and after correction are illustrated in Figure 16, and the distress ratios are summarized in Table 6. In Figure 16, the red contour represents the standard marking contour obtained through direct matching, while the green contour denotes the corrected contour derived from the contour correction method. The results indicate that the contour correction method enhances the alignment between the matched contour and the actual extracted marking contour. This approach effectively mitigates errors caused by both matching inaccuracies and construction deviations.

**Figure 17:**
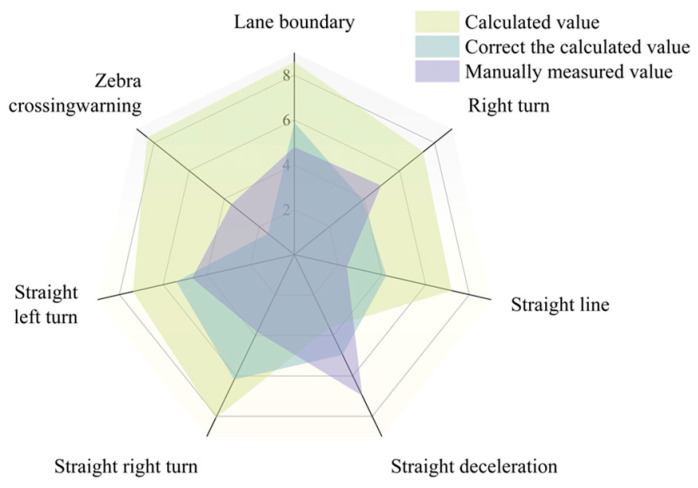
Three methods to obtain the damage rate.

**Figure 18:**
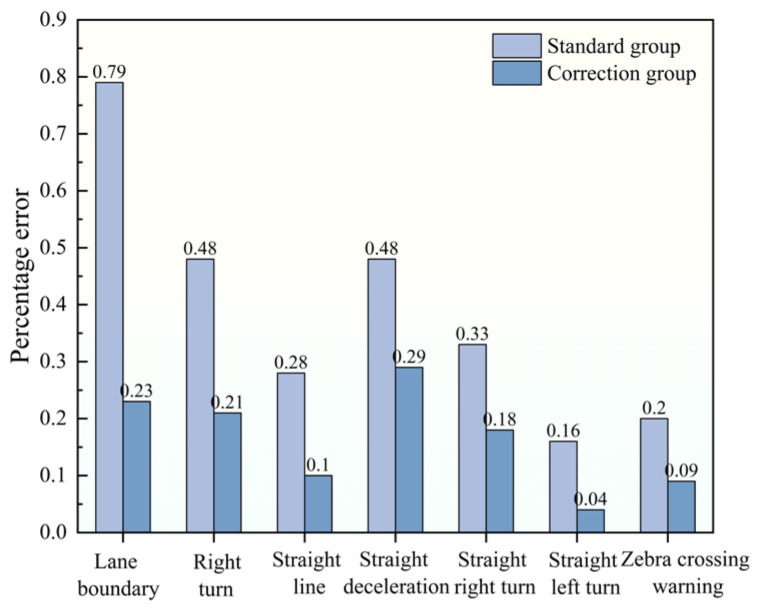
Percentage error chart of non-correction method and correction method.

This study used the proposed framework to detect the damage status of three road markings, and verified the degree of damage of some markings by combining the measured data of manual close-range measurement. Evaluation indicators based on the area of markings were adopted. A comparison between the framework’s evaluation results and manual measurements is shown in Figure 17. Furthermore, Figure 18 presents a comparison of the percentage errors in distress ratio calculations with and without the dynamic contour correction method. As observed in Figure 18, the dynamic contour correction method contributes positively to reducing calculation errors in the distress ratio.

However, the study reveals higher distress ratio errors for linear markings, primarily due to two factors: frequent rolling and wear from vehicle traffic, and their higher perimeter-to-area ratio, which increases susceptibility to edge loss and matching deviations.

**Figure 19:**
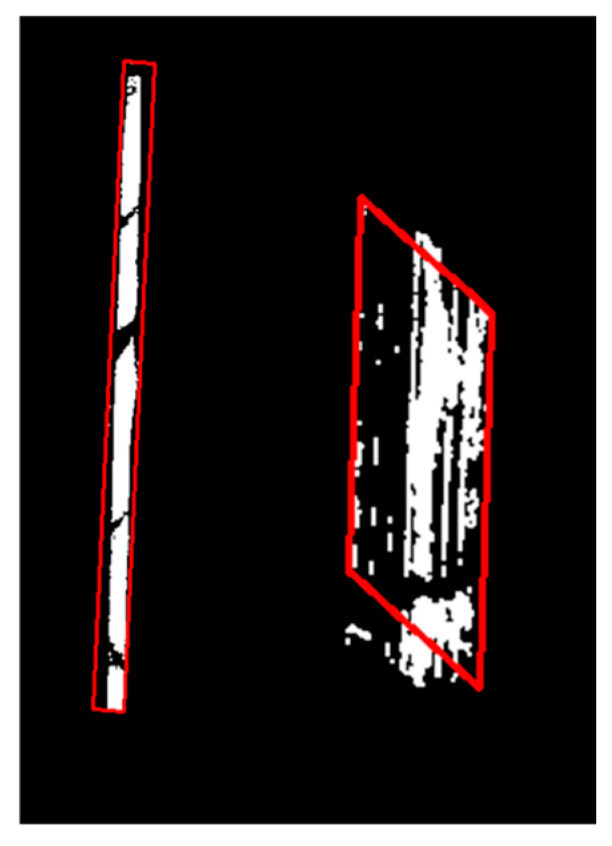
Wrong processing result.

As illustrated in Figure 19, markings with smaller areas, high perimeter-to-area ratios, and frequent traffic exposure are more prone to edge loss. Severe morphological deformations cause significant discrepancies in the aspect ratios of the minimum bounding rectangles between the actual markings and the standard templates, resulting in matching failures. Based on a comprehensive analysis of the data in this study, the average error of the calculated damage rate using the proposed framework can be controlled within 10% for non-severe damage (damage rate < 26.4%). However, its applicability decreases significantly when dealing with severe damage cases (damage rate > 50%). In such scenarios, due to the combined errors from the detection model and the RANSAC matching model, the final assessment results typically exhibit an error exceeding 30%. The above data represents an evaluation on the comprehensive dataset. Higher accuracy is achieved for cases where the exterior of the markings is well-preserved (e.g., internal wear, cracking), while higher errors occur for cases with severe damage to the external contours (e.g., edge loss in linear markings). This is because RANSAC relies on a sufficient number of reliable inliers for stable affine transformation estimation. Under severe erosion, sparse and noisy edge points become the dominant outliers, leading to unstable model fitting, template matching errors, and an overestimation of the damage rate.

This limitation restricts the general applicability of the method primarily to the active monitoring and early maintenance of road networks, rather than to the comprehensive assessment of severely damaged aging infrastructure. Meanwhile, to ensure generality, the experiments in this study only used image data for analysis. However, for GPS-equipped UAVs capable of acquiring relative altitude, the detection error can be significantly reduced by inputting the relative altitude to determine the GSD of the image.

## 4. Conclusions

Clear road markings are essential infrastructure for ensuring traffic safety and operational efficiency. In the context of autonomous driving applications, high-quality markings provide critical support for vehicle perception systems in lane recognition, precise positioning, and path planning. Existing research has primarily focused on the identification and detection of road markings, while systematic methods for quantitatively assessing their distress conditions remain underdeveloped. To address this gap, this study proposes a comprehensive framework for road marking distress detection and evaluation based on UAV imagery, covering the entire process from data acquisition and marking extraction to distress quantification. By optimizing UAV flight parameters and inspection routes, efficient and lightweight image acquisition was achieved. During the marking extraction stage, a two-level architecture integrating initial instance segmentation and local refinement was constructed, effectively suppressing environmental interference such as global shadows and water accumulation, with an extraction accuracy exceeding 90%. In the evaluation phase, a distress quantification system for multiple types of markings was designed. By reconstructing the intact morphology of markings and incorporating a dynamic contour correction strategy, the impact of construction deviations on evaluation results was mitigated. The evaluation error of conventional damage markers should be controlled within 10%. Future research will expand the dataset with greater geographic diversity and scale, incorporate rigorous statistical analysis and robustness testing under varied UAV flight conditions (e.g., altitude, lighting, weather), and conduct comparisons with alternative UAV-based pavement distress assessment frameworks to further validate and enhance the proposed method’s reliability and real-world applicability.

## Figures and Tables

**Table 1:** UAV and camera equipment parameters.

Key Parameters	Parameter Value
Equipment combination	DIJ M300 RTK and ZH20N
Sensor size	1/1.8″CMOS
Focal length	6.77–119.9 mm
Aperture size	f/1.6–f/11
Max shutter speed	1/8000
Max wind resistance level	15 m/s (Force 7 winds)
Max endurance time	55 min
Max takeoff weight	9 kg

**Table 2:** Minimum flight altitude table corresponding to the number of lanes. (a) Twice the focal length (12.15 mm). (b) Four the focal length (24.25 mm).

(a)			
**Number of Lanes**	**Road Width (m)**	**Corrected View Width (m)**	**Minimum Flight Height (m)**
1	3.75	4.17	7.12
2	7.5	8.33	14.24
3	11.25	12.5	21.36
4	15	16.67	28.48
5	18.75	20.83	35.6
6	22.5	25	42.72
(b)			
**Number of Lanes**	**Road Width (m)**	**Corrected View Width (m)**	**Minimum Flight Height (m)**
1	3.75	4.17	15.05
2	7.5	8.33	30.01
3	11.25	12.5	45.14
4	15	16.67	60.19
5	18.75	20.83	75.24
6	22.5	25	90.29

**Table 3:** Operating environment related parameters.

Type	Parameter Value
Operating system	Windows 10
Graphics card	NVIDIA GeForce RTX 2060 Ti
Memory	16 GB
Development environment	Pycharm 2023.2.2
Cuda	Cuda 12.1
Programming language	Python 3.8

**Table 4:** YOLOv8-MEB ablation experiment.

Model	P	R	mAP@0.5	mAP@0.5:0.95
YOLOv8-MobileNetV4	92.6%	96.7%	94.5%	81.4%
YOLOv8-MB	94.7%	97.5%	96.2%	85.3%
YOLOv8-ME	94.9%	96.9%	97.2%	84.6
YOLOv8-MEB	95.5%	98.3%	98.7%	87.3%

**Table 5:** Compared with other models.

Model	Box/mAP	Mask/mAP	Parameters	FPS
Mask R-CNN [51]	83.6%	85%	32 M	8
YOLO-ShuffleNetv2 [52]	84.1%	83.8%	3.9 M	68
ASF-YOLO [53]	89.5%	88.8%	46 M	21
YOLACT [54]	83.2%	82.5%	36 M	15
YOLOv5seg	84.9%	84.1%	7.4 M	31
YOLOv8seg [55]	86.8%	85.8%	3.4 M	48
Ours	86.1%	85.2%	5 M	72

**Table 6:** Comparison of damage rate calculation standard group and correction group.

Group	1	2	3	4	5	6
Standard group	8.5%	2.2%	22.1%	6.5%	14.29%	35.9%
Correction group	7.4%	2.3%	23.8%	7.1%	13.8%	35.6%

## Data Availability

The original contributions presented in this study are included in the article. Further inquiries can be directed to the corresponding authors.
